# An evaporite sequence from ancient brine recorded in Bennu samples

**DOI:** 10.1038/s41586-024-08495-6

**Published:** 2025-01-29

**Authors:** T. J. McCoy, S. S. Russell, T. J. Zega, K. L. Thomas-Keprta, S. A. Singerling, F. E. Brenker, N. E. Timms, W. D. A. Rickard, J. J. Barnes, G. Libourel, S. Ray, C. M. Corrigan, P. Haenecour, Z. Gainsforth, G. Dominguez, A. J. King, L. P. Keller, M. S. Thompson, S. A. Sandford, R. H. Jones, H. Yurimoto, K. Righter, S. A. Eckley, P. A. Bland, M. A. Marcus, D. N. DellaGiustina, T. R. Ireland, N. V. Almeida, C. S. Harrison, H. C. Bates, P. F. Schofield, L. B. Seifert, N. Sakamoto, N. Kawasaki, F. Jourdan, S. M. Reddy, D. W. Saxey, I. J. Ong, B. S. Prince, K. Ishimaru, L. R. Smith, M. C. Benner, N. A. Kerrison, M. Portail, V. Guigoz, P.-M. Zanetta, L. R. Wardell, T. Gooding, T. R. Rose, T. Salge, L. Le, V. M. Tu, Z. Zeszut, C. Mayers, X. Sun, D. H. Hill, N. G. Lunning, V. E. Hamilton, D. P. Glavin, J. P. Dworkin, H. H. Kaplan, I. A. Franchi, K. T. Tait, S. Tachibana, H. C. Connolly, D. S. Lauretta

**Affiliations:** 1 Department of Mineral Sciences, National Museum of Natural History, Smithsonian Institution, Washington, DC USA; 2 Planetary Materials Group, Natural History Museum, London, UK; 3 Lunar and Planetary Laboratory, University of Arizona, Tucson, AZ USA; 4 Jacobs Technology, NASA Johnson Space Center, Houston, TX USA; 5 Schwiete Cosmochemistry Laboratory, Goethe University, Frankfurt, Germany; 6 Space Technology and Science Centre, School of Earth and Planetary Sciences, Curtin University, Perth, Western Australia Australia; 7 John de Laeter Centre, Curtin University, Perth, Western Australia Australia; 8 Université Côte d’Azur, Observatoire de la Côte d’Azur, CNRS, Laboratoire Lagrange, Nice, France; 9 Space Sciences Laboratory, University of California, Berkeley, CA USA; 10 Department of Physics, California State University, San Marcos, CA USA; 11 Astromaterials Research & Exploration Science (ARES), NASA Johnson Space Center, Houston, TX USA; 12 Department of Earth, Atmospheric, and Planetary Sciences, Purdue University, West Lafayette, IN USA; 13 NASA Ames Research Center, Moffett Field, CA USA; 14 Department of Earth and Environmental Sciences, The University of Manchester, Manchester, UK; 15 Natural History Sciences, Hokkaido University, Sapporo, Japan; 16 Department of Earth and Environmental Sciences, University of Rochester, Rochester, NY USA; 17 Lawrence Berkeley National Laboratory, Berkeley, CA USA; 18 School of the Environment, University of Queensland, St Lucia, Queensland Australia; 19 Creative Research Institution, Hokkaido University, Sapporo, Japan; 20 Université Côte d’Azur, CNRS, CRHEA, Valbonne, France; 21 CNRS, Université Jean Monnet Saint-Étienne, Saint-Etienne, France; 22 Southwest Research Institute, Boulder, CO USA; 23 NASA Goddard Space Flight Center, Greenbelt, MD USA; 24 Faculty of Science, Technology, Engineering & Mathematics, Open University, Milton Keynes, UK; 25 Department of Natural History, Royal Ontario Museum, Toronto, Ontario Canada; 26 Department of Earth and Planetary Sciences, University of Tokyo, Tokyo, Japan; 27 Department of Geology, Rowan University, Glassboro, NJ USA; 28 Department of Earth and Planetary Sciences, American Museum of Natural History, New York, NY USA; 29 Present Address: Los Alamos National Laboratory, Los Alamos, NM USA

**Keywords:** Mineralogy, Asteroids, comets and Kuiper belt, Astrobiology, Petrology, Early solar system

## Abstract

Evaporation or freezing of water-rich fluids with dilute concentrations of dissolved salts can produce brines, as observed in closed basins on Earth^1^ and detected by remote sensing on icy bodies in the outer Solar System^2,3^. The mineralogical evolution of these brines is well understood in regard to terrestrial environments^4^, but poorly constrained for extraterrestrial systems owing to a lack of direct sampling. Here we report the occurrence of salt minerals in samples of the asteroid (101955) Bennu returned by the OSIRIS-REx mission^5^. These include sodium-bearing phosphates and sodium-rich carbonates, sulfates, chlorides and fluorides formed during evaporation of a late-stage brine that existed early in the history of Bennu’s parent body. Discovery of diverse salts would not be possible without mission sample return and careful curation and storage, because these decompose with prolonged exposure to Earth’s atmosphere. Similar brines probably still occur in the interior of icy bodies Ceres and Enceladus, as indicated by spectra or measurement of sodium carbonate on the surface or in plumes^2,3^.

## Abstract (web summary)

Samples from the asteroid (101955) Bennu, returned by the OSIRIS-REx mission, include sodium-bearing phosphates and sodium-rich carbonates, sulfates, chlorides and fluorides formed during evaporation of a late-stage brine.

## Main

Brines (over 3.5 wt% dissolved solids) are environments in which life could have evolved or might persist in the Solar System^6^, and are targets for spacecraft exploration. Evaporation or freezing can lead to the formation of brines from which a variety of minerals (for example, carbonates, sulfates and halides) precipitate. On Earth, such mineral deposits are a major source of technologically critical elements^7^. On Mars, brine freezing points extend to approximately −20 °C, prolonging the liquid state of water^8^. Icy outer Solar System bodies contain subsurface brines, sometimes as oceans. Evidence of subsurface brines is found on Saturn’s moon Enceladus^9^ and the dwarf planet Ceres^3^, the largest body in the asteroid belt.

Our knowledge of brines beyond Earth is hampered by a lack of samples. Remote-sensing observations of Mars, Ceres and Enceladus limit our ability to determine precipitated phases in minor to trace abundances, unravel the age and timing of fluid evolution and precipitation and determine the compositions of the associated fluids. Evaporite phases known from meteorites are extremely limited. These include sulfates and halides in Martian nakhlites^10^, intrusive igneous rocks that experienced secondary alteration, and potentially indigenous halite in ordinary chondrites^11^.

Early analyses of samples from Bennu recorded evidence of pervasive aqueous alteration—including hydrated phyllosilicate clay minerals, carbonates, magnetite and sulfides^5^—similar to samples from asteroid (162173) Ryugu returned by the Hayabusa2 mission and the most extensively aqueously altered carbonaceous chondrites (for example, Ivuna-type (CIs)). Pervasive aqueous alteration did not occur on Bennu itself, which is a second- or later-generation rubble pile formed within the past 65 million years^12^, but on Bennu’s larger parent asteroid in the early Solar System^13^. Remote sensing of Bennu by the OSIRIS-REx mission showed distinct boulder populations that probably formed at different depths within the parent body^14,15^ and were probably sampled by the spacecraft^5,16,17^.

**Fig. 1:**
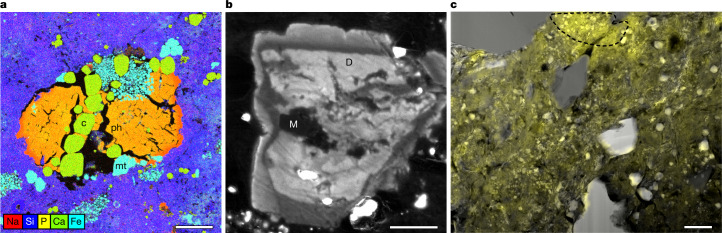
Carbonate occurrences and textures in Bennu samples. **a**, Multiphase particle exhibiting successive growth of calcite (c), magnetite (mt) and zoned Mg,Na phosphate (ph), set in a matrix of phyllosilicates (multielement X-ray map; sample no. OREX-803065-0). **b**, HR-CL image of zoned carbonate. A core of Fe,Mn-bearing magnesite (M) appears dark (non-luminescent) and is surrounded by multiple dolomite overgrowths (D) exhibiting a range of luminescence intensity (dull to bright) around 650 nm due to Mn variations. Phase identification confirmed by SEM (sample no. OREX-800045-106). **c**, X-ray map of molecular carbonate acquired using scanning transmission X-ray microscopy by integration of the area under the 290.5 eV peak (yellow) overlaid on an inverted bright-field TEM image of sample no. OREX-803030-100, demonstrating that molecular carbonate is heterogeneous and ubiquitous in phyllosilicates. The spectrum of the outlined area is shown in Extended Data Fig. 3. Scale bars, 20 μm (**a**), 5 μm (**b**) and 2 μm (**c**).

**Extended Data Fig. 1:**
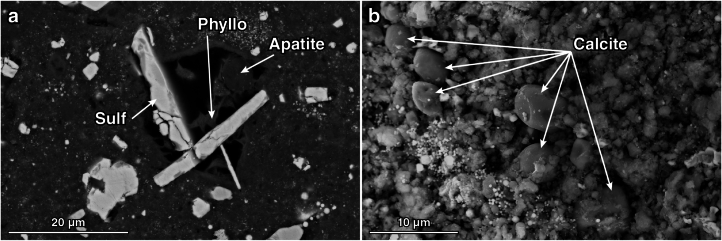
Early-forming phases during water-rock interactions on Bennu. (a) Subhedral void with co-existing sulfide laths/blades, apatite, and fibrous phyllosilicates (BSE image; OREX-501062-100). (b) Calcites of near–end member composition occur often as ovoid shapes resembling terrestrial scalenohedral calcite crystals (BSE image; OREX-501019-0).

**Extended Data Fig. 2:**
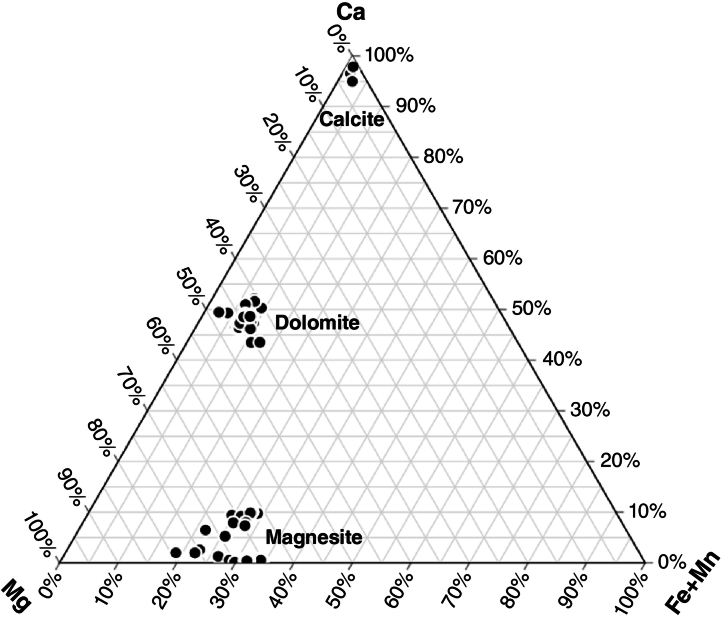
Ca,Mg,Fe,Mn carbonate compositions determined by SEM EDS and EMPA wavelength-dispersive spectrometry. Compositions are plotted in a ternary diagram of Ca-Mg-Fe+Mn, illustrating groupings of near–end member calcite, dolomite with minor Fe and Mn, and Fe,Mn-bearing magnesite (OREX-803071-0, OREX-803085-100, OREX-803090-100, OREX-803098-100, OREX-803102-104, OREX-803102-105).

The Bennu samples contain an array of phases formed by precipitation from a fluid. The most abundant of these phases include sulfides, magnetite and carbonates (10, 5 and 3 vol.%, respectively, by X-ray diffraction of a bulk sample^5^), although concentrations differ between individual millimetre-sized asteroidal particles. These phases often occur in association. Sulfides, primarily pyrrhotite, occur as pseudohexagonal mineral grains tens to hundreds of microns in size, micron-size anhedral grains^5^ and vug-filling laths, the last of these being associated with magnesian phyllosilicates and apatite^5^ (Extended Data Fig. 1a). Carbonate includes grains of calcite (CaCO_3_), dolomite ((Mg,Ca)CO_3_) and Fe,Mn-bearing magnesite (MgCO_3_). Using scanning electron microscopy (SEM), we observed subhedral to rounded calcite occurring with magnetite (Fe_3_O_4_), the latter as framboids (Fig. 1a), plaquettes and spherules. These phases also appear separately. Calcite occurs as ovoid shapes with aspect ratio of roughly 2:3 (Extended Data Fig. 1b), and magnetite framboids occur in veins. SEM and electron microprobe analysis (EMPA) show that the calcite has near-end member composition (4 mol.% or less MgCO_3_ and FeCO_3_; 0.1 mol.% or less MnCO_3_; Extended Data Fig. 2).

Intergrowths of Ca,Mg,Fe,Mn-bearing carbonates, including dolomite and Fe,Mn-bearing magnesite, are observed using SEM and high-resolution cathodoluminescence (HR-CL). Composite grains exhibit mixed cores of Fe,Mn-bearing magnesite and dolomite, sometimes rimmed by subsequently precipitated dolomite (Fig. 1b). Dolomite also occurs as veins cross-cutting the phyllosilicate matrix and 200–500-µm grains as the dominant phase at millimetre scale. SEM and EMPA indicate dolomite of subequal molar Ca and Mg, with minor Fe and Mn (under 11 mol.% FeCO_3_, under 8 mol.% MnCO_3_; Extended Data Fig. 2). Fe,Mn-bearing magnesites are Mg dominant, with under 33 mol.% FeCO_3_, under 18 mol.% MnCO_3_ and under 10 mol.% CaCO_3_ (Extended Data Fig. 2).

**Extended Data Fig. 3:**
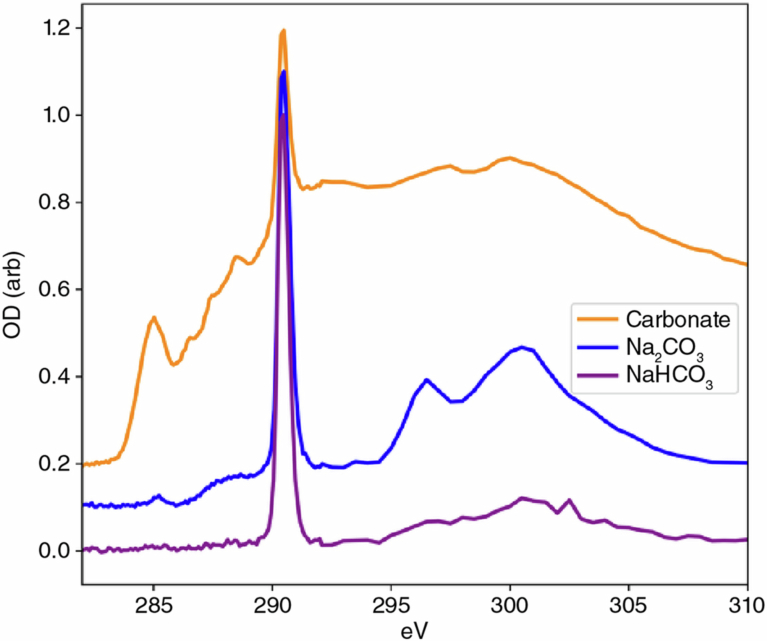
STXM spectrum (orange) of a carbonate-rich region of phyllosilicate matrix . STEM EDS shows that the outlined region in Fig. 1c contains abundant carbon, 0.5 at.% Na, and detectable K. The between 295 and 305 eV shows features not typical of pure organics but consistent with carbonates. The Na_2_CO_3_ (blue) and NaHCO_3_ (purple) standards were prepared by allowing ~1 μL of dissolved powder in deionized water to evaporate on a TEM grid. The centroid of the carbonate peak is at 290.50 for Na_2_CO_3_, 290.47 for NaHCO_3_, and 290.46 for Bennu showing excellent experimental agreement.

In addition, we observed a continuum of submicron amorphous to molecular carbonate occurring with phyllosilicates (Fig. 1c) using transmission electron microscopy (TEM) and scanning transmission X-ray microscopy. This carbonate exhibits the 290.5-eV peak characteristic of the carbonate functional group (Extended Data Fig. 3), but does not diffract as crystals on TEM. Similar amorphous carbonate was observed in Ryugu samples^18,19^. This carbonate could represent an important reservoir of total carbonate ions in Bennu samples, given the abundance of phyllosilicates (about 80 vol.%)^5^.

**Fig. 2:**
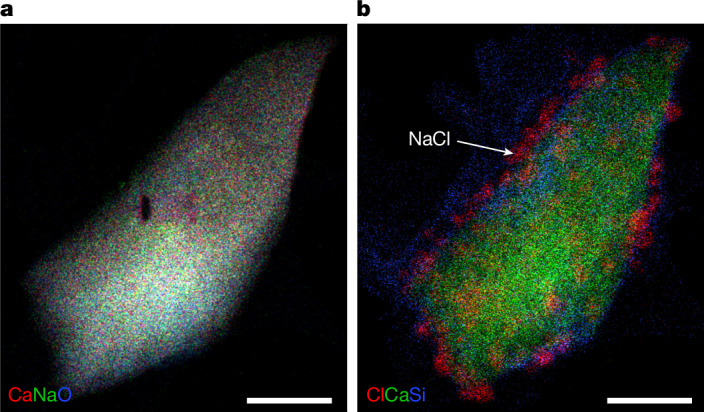
Hydrated Na,Ca carbonate. **a**, Na,Ca carbonate grain, studied about 1 month following removal from nitrogen storage in the curatorial facility, exhibits SAED *d*-spacing consistent with the hydrated phases gaylussite or pirssonite. The mineral is sensitive to beam damage (dark spot within grain), consistent with a hydrated phase (multielement Kα X-ray map; sample no. OREX-800045-102). **b**, NaCl particles grown on the same grain after 5 months (multielement Kα X-ray map). Scale bars, 200 nm.

Nanometre- to micron-sized Na,Ca carbonate grains were studied using TEM (Fig. 2a), including selected-area electron diffraction (SAED). The phase is sensitive to beam damage. Co-occurring phases include calcite, TiO_2_, pyrrhotite, pentlandite and phyllosilicates. Na,Ca carbonate studied by SAED around 1 month following sample allocation yielded *d*-spacings consistent with hydrated Na,Ca carbonates gaylussite (Na_2_Ca(CO_3_)_2_·5H_2_O) or pirssonite (Na_2_Ca(CO_3_)_2_·2H_2_O) (Extended Data Table 1). The 2.84-Å *d*-spacing is inconsistent with anhydrous Na,Ca carbonate, nyerereite (Na_2_Ca(CO_3_)_2_), suggesting that the Bennu Na,Ca carbonate is hydrated. The same grain examined roughly 5 months later following continuous storage in an air-filled desiccator showed extensive alteration, including NaCl growth on the surface (Fig. 2b), suggesting that the phase is highly reactive in air.

**Fig. 3:**
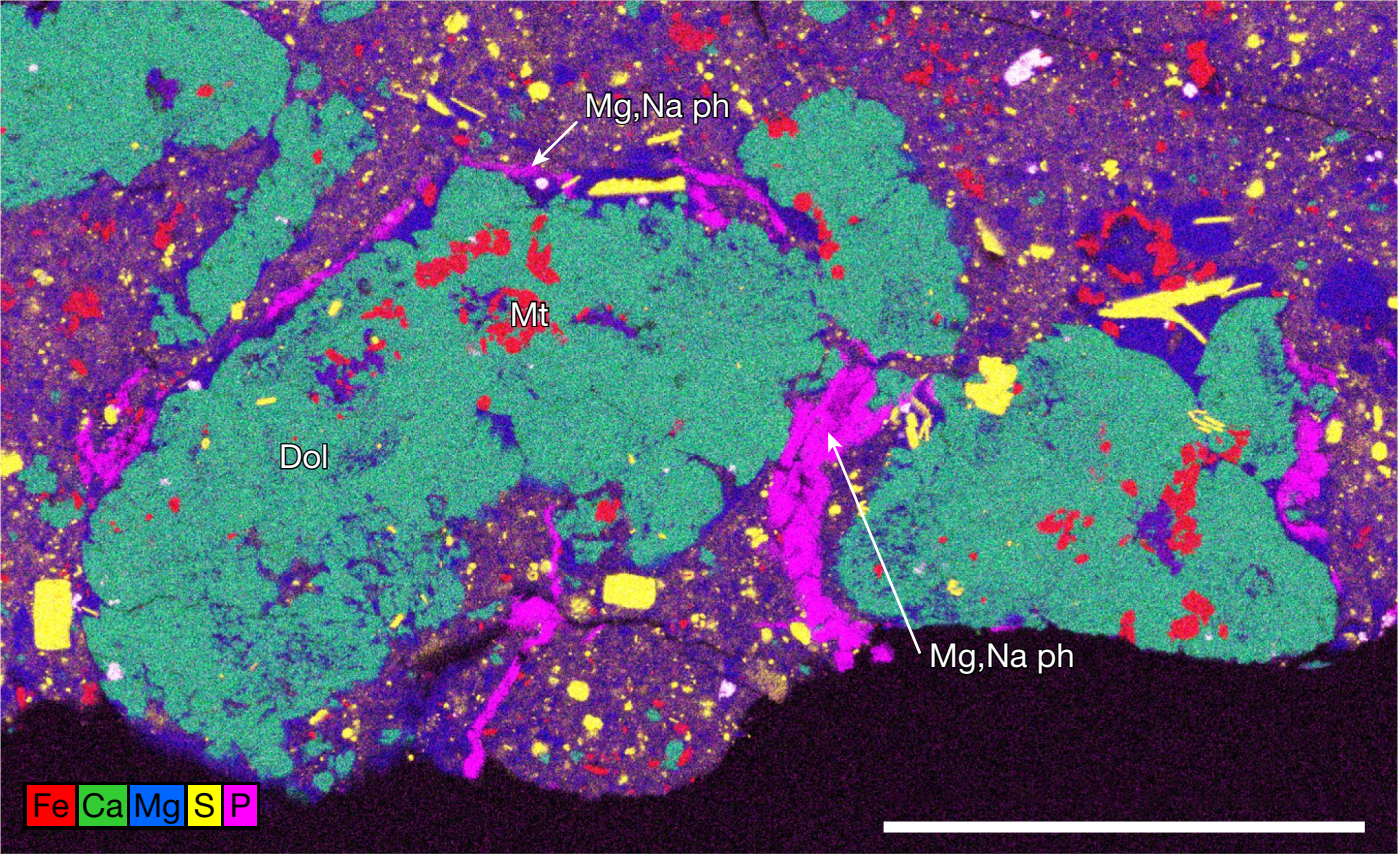
Mg,Na phosphate occurs as sheets and veins. Mg,Na phosphate occurs with Mg phyllosilicates largely free of micron-sized sulfide inclusions. The Mg,Na phosphate–Mg phyllosilicate veins rim large dolomite (Dol) grains that include magnetite (multielement Kα X-ray map; sample no. OREX-803085-100). Scale bar, 200 μm.

**Extended Data Fig. 4:**
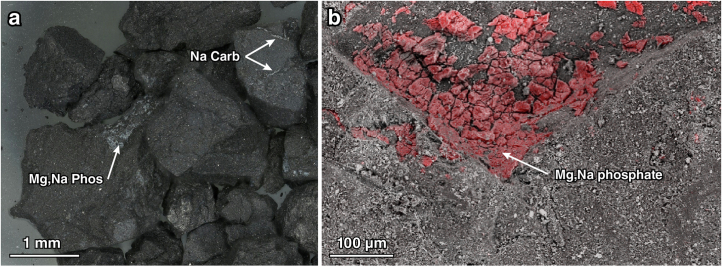
Mg,Na phosphate and Na carbonate in millimeter-sized particles. (a) Optical image of aggregate sample OREX-803013-0, from which Mg,Na phosphate–bearing sample OREX-803085-0 and Na carbonate–bearing sample OREX-803088-0 were separated. The Mg,Na phosphate and Na carbonate are both light-toned and not readily distinguished at this scale. (b) Mg,Na phosphate occurs as broken, blocky grains along a three-dimensional surface indicative of a non-linear vein structure (BSE image with semi-transparent P Kα X-ray map overlay; OREX-803085-0).

**Extended Data Fig. 5:**
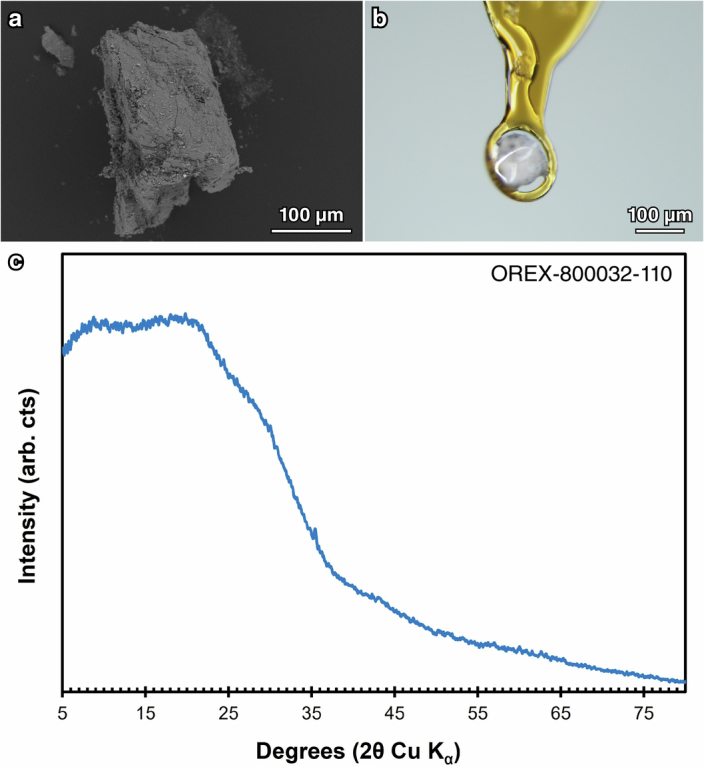
X-ray diffraction demonstrates that Mg,Na phosphate is poorly crystalline or amorphous. (a) SEM BSE image of Mg,Na phosphate OREX-800032-110 mounted on a carbon adhesive disc. (b) Optical image of the particle fixed to a Kapton loop using a small amount of glue. (c) Micro-XRD pattern acquired from the same particle showing the lack of coherent diffraction. A small diffraction peak at ~35° (2θ) may be related to matrix material adhering to the surface of the particle.

Magnesium sodium phosphate forms bright coatings on some Bennu particles^5^ (Extended Data Fig. 4a). It occurs as blocky grains with textures indicative of desiccation. Using X-ray diffraction (XRD), we show that Mg,Na phosphate in Bennu samples is amorphous (Extended Data Fig. 5), probably the result of desiccation^5^. Mg,Na phosphate occurs as a late-stage phase in calcite–magnetite-rich vugs (Fig. 1a); on irregular broken surfaces, suggesting complex three-dimensional vein shapes (Extended Data Fig. 4b); and associated with veins of Mg phyllosilicates lacking micrometre-sized sulfide inclusions that rim large dolomites (Fig. 3). Using SEM energy-dispersive X-ray spectroscopy (EDS), we observed up to 10 wt% Na, although typically 0–5 wt%. Some grains exhibit approximate Na zonation of 2 wt% (Fig. 1a). Minor concentrations of K (0.06 wt% or less), Cl (0.13 wt% or less) and F (0.60 wt% or less) are detected by EMPA.

**Fig. 4:**
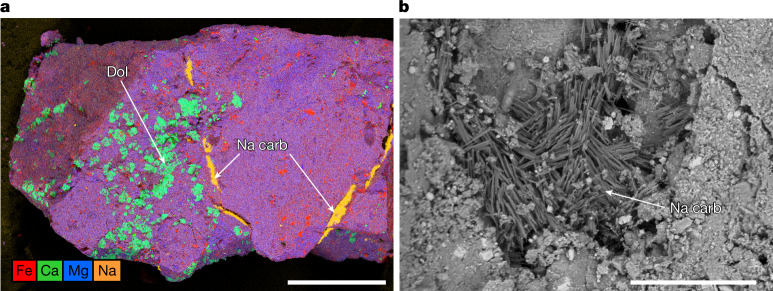
Na carbonate vein or sheet. **a**, Na carbonates (orange) underlie a flat surface, which is itself overlain by dolomite-bearing (teal), phyllosilicate-rich (purple) host rock containing magnetite and sulfide (red) (multielement Kα X-ray map; sample no. OREX-803088-0). **b**, Na carbonate, occuring as needles of less than 1-µm wide and 5–10-µm long, in a vein or sheet that underlies a clast (backscattered electron (BSE) image; sample no. OREX-803088-0). Scale bars, 500 μm (**a**) and 20 μm (**b**).

**Extended Data Fig. 6:**
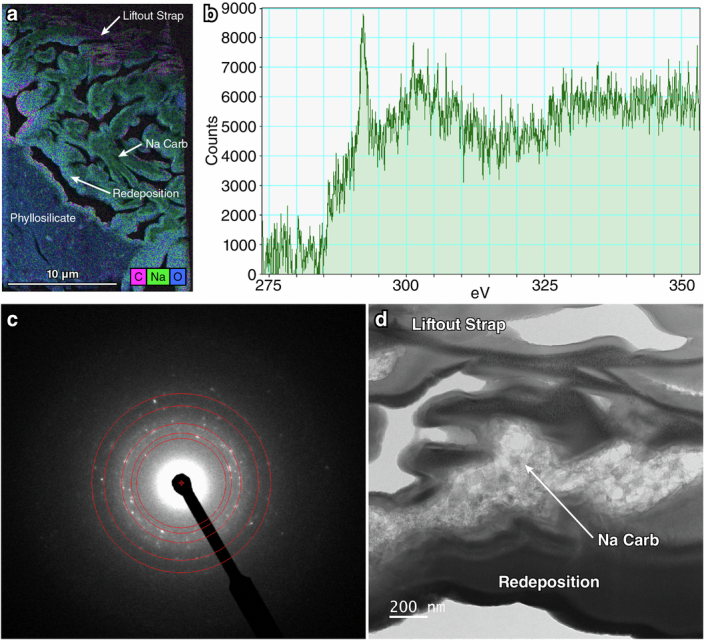
TEM data on Na carbonate. (a) EDS map of FIB liftout section. Na carbonate is rimmed by redeposited material from the FIB sectioning. (b) EELS spectrum of Na carbonate in (d). The sharp peak at ~290 eV in the carbon K edge is consistent with the presence of carbonate groups. (c) Selected area diffraction pattern of Na carbonate in Fig. 4d with lines indexed by radial integration of the scattering. The pattern largely consists of spots and short arcs due to the nanocrystalline nature of the material. (d) Bright-field TEM imaging showing spongy, lacy Na carbonate rimmed by material redeposited during preparation of the FIB section and capped by carbon from the liftout strip from FIB sectioning.

Late-stage, sodium-rich phases typically occur as isolated veins or individual grains, rather than being associated with other salts. Veins of Na carbonate up to 20 µm in width and 200 µm in length form segments, possibly exposures of a continuous sheet, bounding a clast (Fig. 4a and Extended Data Fig. 4). Within these veins, Na carbonate occurs as randomly oriented needles of width below 1 µm and length 5–10 µm (Fig. 4b). Both SEM and TEM show that this phase is beam sensitive, probably reflecting the hydrated nature of the phase, and is composed of Na, C and O (Extended Data Fig. 6a). In TEM bright-field imaging, Na carbonate has a highly porous and lacy texture (Extended Data Fig. 6d), possibly reflecting, in part, damage due to focused ion beam (FIB) milling. Scanning TEM (STEM)-based electron energy-loss spectroscopy (EELS) mapping shows a sharp peak at around 290 eV in the carbon K edge (Extended Data Fig. 6b), consistent with carbonate groups. SAED (Extended Data Fig. 6c and Extended Data Table 2) from several locations shows that the Na carbonate is poorly crystalline. Matches for carbonates with allowable Na, C, O and H and measured *d*-spacings include the hydrous phase trona (Na_3_H(CO_3_)2·2H_2_O) and the H-bearing phases wegscheiderite (Na_5_H_3_(CO_3_)_4_) and nahcolite (NaHCO_3_). Wegschiederite is the poorest fit to *d*-spacings and trona the best, although the nanocrystalline nature increases uncertainty. We favour identification as trona but cannot exclude nahcolite or a mixture of the two.

**Fig. 5:**
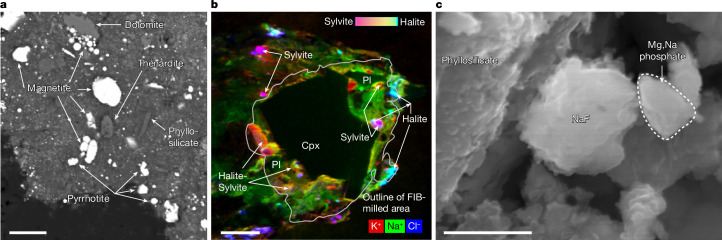
Na-rich evaporite phases. **a**, Na sulfate, probably thénardite, occurs as multiple grains (roughly 5 µm) in a phyllosilicate-dominated clast (BSE image; sample no. OREX-803172-0). **b**, Halite and sylvite occur in a particle with calcic pyroxene (Cpx) and plagiolcase (Pl) (ToF–SIMS ion map; sample no. OREX-501070-0). **c**, Sodium fluoride grains of approximately 1 µm, possibly villiaumite, occur with phyllosilicates and Mg,Na phosphate (secondary electron image; sample no. OREX-803174-0). Scale bars, 10 μm (**a**), 5 μm (**b**) and 1 μm (**c**).

Sodium sulfate occurs as distinct grains of roughly 5 µm within a single particle dominated by phyllosilicates (Fig. 5a). The composition of sodium sulfate by EDS is 32.8 wt% Na, 19.2 wt% S and 48 wt% O, yielding a formula of Na_1.9_S_0.8_O_4_, a reasonable approximation for thénardite (Na_2_SO_4_).

**Extended Data Fig. 7:**
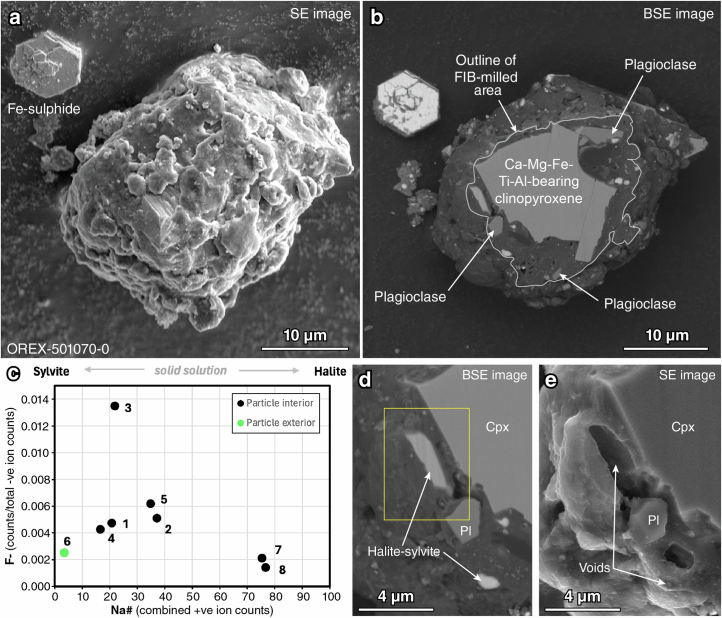
Halite and sylvite grains in Bennu sample OREX-501070-0. (a) SE image of halite- and sylvite-bearing particle subjected to FIB milling. (b) BSE image of the particle in (a) after FIB-milling the top 10 µm, revealing Ca-Mg-Fe-Ti-Al–bearing clinopyroxene, plagioclase, halite, and sylvite in a Na- and C-rich matrix. (c) Plot of F versus Na# of the halite-sylvite fragments. (d) BSE image zoomed in on the halite and sylvite. (e) SE image collected after 8 months, showing voids where halite-sylvite grains were once located.

Halite (NaCl) and sylvite (KCl) were observed as angular to rounded grains smaller than 5 μm (mostly under 1 μm) in size, co-existing with calcic pyroxene and plagioclase in a Na-rich phyllosilicate matrix (Fig. 5b and Extended Data Fig. 7). Time-of-flight–secondary ion mass spectrometry (ToF–SIMS) indicates variable Na and K between grains. Both phases contain detectable, although variable, F. Reimaging of the particle after 8 months in an air-filled autodesiccator showed that halite-sylvite grains had disappeared, leaving voids (Extended Data Fig. 7e).

Sodium fluoride occurs as rare grains roughly 1 µm in size, co-existing with phyllosilicate matrix and Mg,Na phosphate (Fig. 5c). The composition of sodium fluoride by EDS is 40.7 wt% F and 59.3 wt% Na, corresponding to a formula of Na_1.22_F. No Cl was detected. Given the uncertainties in analysis of small, irregular grains, this is a reasonable approximation for villiaumite (NaF).

The occurrence of diverse salts, particularly Na-rich salts, distinguishes Bennu samples from meteorites, in which evaporite minerals are rare. Extraterrestrial halite and sylvite have been reported in both ordinary chondrites^11^ and aqueously altered Mighei-type chondrites^20^. Hydrated Na sulfate (for example, bloedite, MgSO_4_·Na_2_SO_4_·4H_2_O (ref. ^21^)) in the CI chondrite Ivuna was attributed to terrestrial alteration. Surficial sulfates and halite formed in the Mighei-type chondrite Winchcombe days to months after its fall^22^. These observations support the idea that extensive sulfate veining in CI chondrites, once attributed to asteroidal aqueous alteration, formed by terrestrial alteration^23^—as does the alteration of Na,Ca carbonate (Fig. 2b) and complete loss of NaCl and KCl (Extended Data Fig. 7e) in Bennu samples stored in air-filled desiccators over 5–8 months at relative humidity of around 10–40%.

Volatile element depletions observed in chondrites^24^ that have had residence times on Earth from years to centuries could be explained in part by loss of evaporites rich in Na, Cl, F and S due to prolonged contact with air and attendant water vapour. By contrast, Bennu samples stored in nitrogen (much less than 1% relative humidity) retained their Na-rich evaporite phases, including Na sulfate, carbonate and fluoride. In the absence of sample return, curation and storage during analysis under carefully controlled conditions (in nitrogen), this full array of salts would not have been discovered.

The array of salts and associated phases in Bennu samples suggests that a protracted fluid evolution occurred on Bennu’s parent asteroid, notably forming veins that cross-cut the host rock. On Earth, these salts form primarily by precipitation from fluids. Closed basins serve as the optimal terrestrial analogue for Bennu’s parent body, which would have undergone limited or no input of fluid during evaporation or freezing. In terrestrial closed basins, dilute solutions can produce brines through evaporation or freezing^1^, and the nature of those brines is controlled largely by mineral precipitation events, termed chemical divides. Carbonate precipitation has been suggested to produce fluids rich in Na, SO_4_ and Cl, and poor in Mg and Ca^25,26^.

Among the best-studied terrestrial basins with early Ca,Mg carbonate precipitation is Searles Lake in California, USA^4,27^. Calcite and dolomite formed there by precipitation primarily because of initial saturation of the fluid, rather than evaporation. Na,Ca carbonate (gaylussite/pirssonite) at Searles Lake, formed during syndepositional back-reaction between previously formed carbonate and Na-rich brine, was the last phase to precipitate before evaporation dominated. Later phases in the stratigraphic sequence at Searles Lake, including Na sulfate, Na carbonate and Na chloride, form an evaporite sequence. Similar studies have been conducted for freezing of terrestrial lakes and proposed brines on outer planet moons^28^.

**Fig. 6:**
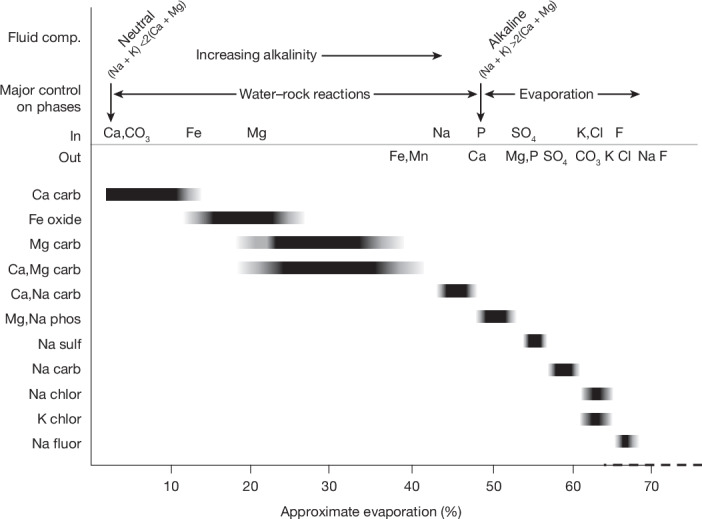
Sequence of mineral phases formed as the brine on Bennu’s parent body evaporated. Black bars indicate the appearance of phases formed by precipitation and evaporation as a function of approximate percentage of brine evaporation, determined by the salinity at which the phase would first appear (Methods). The first and final appearances of a variety of elements, along with evolution from neutral to alkaline, Na-rich fluids, are shown at the top.

Bennu samples contain six of the minerals observed at Searles Lake: calcite, dolomite, gaylussite/pirssonite, thénardite, trona and halite. In the absence of petrographic constraints on the timing of the latter four phases, we adopt the Searles Lake order of formation (Fig. 6). Four salt or associated phases in Bennu samples are not observed at Searles Lake. Abundant magnetite was an early-formed precipitate, as evidenced by magnetite included in dolomite (Fig. 3). Magnetite plaquette and framboid shapes may form by interaction of iron sulfides with an alkaline fluid^29^, evidenced by surficial Na coating magnetite framboids in the Tagish Lake ungrouped carbonaceous chondrite^30^. Coprecipitation of sulfide and apatite, consistent with their co-occurrence in vugs, may have occurred in neutral fluids before calcite precipitation. Together, calcite, dolomite, magnesite, magnetite, apatite and gaylussite/pirssonite were formed during the early stages of water–rock interactions on the parent body.

By contrast, Na-bearing phosphate, Na carbonate, sulfate, chloride, fluoride and K chloride form a late-stage evaporite sequence. Magnesium sodium phosphates probably precipitated in the middle of the mineral sequence, based on relevant terrestrial analogues and their spatial relationships (Figs. 1a and 6). The low-temperature secondary mineral apexite (NaMg(PO_4_)·9H_2_O (ref. ^31^)), from Nevada, USA, occurs with calcite and gaylussite, suggesting a relationship. In Bennu’s parent body, phosphate abundance was probably controlled by the apatite-carbonate buffer^32^, and evaporation increased Na concentrations to the point at which Mg,Na phosphate precipitated. Following precipitation of Na sulfate and Na carbonate, sylvite coprecipitated with halite. Na fluoride probably precipitated after halite when Cl in the fluid was controlled by the solubility of halite. Bennu halides contain tenths of a weight percentage of F, consistent with high F concentration in the fluid. Other classes of minerals observed in closed basins on Earth, including borates and lithium-rich fluorides and brines, are not observed in Bennu samples, consistent with the parts-per-million concentrations of B and Li in their bulk composition^5^.

The salt sequence recorded in the Bennu samples (Fig. 6) may partially constrain the temperature within the parent body, and the relative roles of evaporation and freezing, which could occur in a liquid brine as low as the H_2_O-NH_4_ eutectic (176 K). Modelling of the Searles Lake evaporites constrains temperatures to 20–29 °C (ref. ^4^). Although we have far fewer constraints for Bennu’s parent body (for example, *p*CO_2_, co-occurrence of minerals, initial fluid composition), this temperature range is consistent with maximum formation temperatures for gaylussite (below 40 °C (ref. ^33^)) and pirssonite (below 55 °C (ref. ^34^)) and minimum equilibrium formation temperature for trona (above 20 °C (ref. ^35^)); it is also compatible with temperatures inferred from co-existing sulfide compositions in Bennu samples. We recognize that trona can form by flash-freezing of brines occuring with NaCl^36^, an association not observed in Bennu samples. Together, these geothermometer temperatures favour evaporation, rather than freezing, as the dominant control on fluid composition, at least through the formation of Na carbonate. Fluid freezing following Na carbonate formation is possible.

The setting and relative timing of salt formation can be constrained by the evaporation required. Single-phase evaporite veins suggest precipitation in cracks within Bennu’s parent body. Whether these are small-scale offshoots of a larger brine body is unclear. Preliminary calculations (Methods) suggest formation of Na carbonate and Na chloride at a minimum of 60–70% evaporation. The physical plausibility of extensive evaporation of a fluid moving solely through cracks is unconstrained. By contrast, larger volumes of water, from which evaporating brine could be periodically injected into small fractures, would be consistent with findings from spacecraft observations of Bennu. Sedimentary deposition of large clasts within layered boulders on Bennu would require water several metres deep inside the parent body^37^. Metre-scale bodies of water on Bennu’s parent body are evidenced by veins within boulders on Bennu^38^; spectral features suggest that these veins are primarily carbonates (calcite, dolomite, magnesite) and associated organics. Smaller-scale carbonate veins have been observed in a Bennu sample (Fig. 4 and Extended Data Fig. 4), albeit as Na carbonate, which was not detected on Bennu during proximity operations. This is, perhaps, not surprising given the dominance of Ca,Mg carbonates in Bennu samples. We suggest that carbonate veins on Bennu are metre-scale brine pockets that produced the salts observed and were trapped following global alteration.

Brines are a subject of intense interest to the planetary science community^39^ in regard to understanding the internal structure of ice-rich worlds, and to the formation and preservation of prebiotic organics and their astrobiological potential. Samples returned from the carbonaceous asteroid Ryugu contain Ca,Mg carbonates, Mg,Na phosphate^40^ and Na carbonate^41^. Extracts produced with hot water, termed salts, contain (Na + K/Na + K + Mg + Ca) of about 0.9 and, for formic acid extracts, termed carbonates, this value is roughly 0.10–0.15 (ref. ^42^), suggesting that additional Na-rich evaporite phases may be present in Ryugu samples.

Sodium carbonate (possibly hydrated) and hydrated sodium chloride were spectrally identified in high-albedo areas on Ceres^3,43^. These deposits probably result from recent emplacement of deep-seated brines, perhaps by impact processes^44,45^. Hydrated Na sulfates and carbonates exposed on the surface of an airless body dehydrate in 10^3^–10^8^ years^46^, dependent in part on temperature. The occurrence of Na carbonate on Bennu’s parent body suggests that its ancient brine, at about 55% evaporation, produced evaporites similar to those emplaced recently on Ceres. Furthermore, earlier stages of water–rock interaction and evaporation may have produced abundant Ca,Mg carbonates, magnetite and phosphates on Ceres by analogy with Bennu. Ammonium chloride or ammonium carbonates^3^ observed in bright deposits on Ceres suggest brine temperatures much lower than those for H_2_O/NaCl mixtures. These phases have not yet been observed in Bennu material, but comparatively high concentrations of ammonium (13.61 ± 0.36 µmol g^−^^1^) occur in a hot-water extract of bulk sample^47^.

Active plumes have been observed on Enceladus, with ejected material forming the faint E ring around Saturn^48^. Salt-rich ice grains from these plumes, analysed by Cassini’s Cosmic Dust Analyzer, showed ions of Na, K, Cl, CO_3_ and PO_4_, including Na carbonate^2^ and Na phosphate^32^. On Enceladus, active subsurface brine oceans cryovolcanically erupt materials comparable to those of the ancient evaporites from Bennu’s parent body. Strong scientific justification exists for future missions to Ceres and Enceladus.

The evolving brine and phyllosilicate-rich host rock on Bennu’s parent body pose an intriguing, but untested, environment for prebiotic organic syntheses in a single location. Among the most interesting challenges is the origin of prebiotic organic molecules, particularly nucleotides (nucleobase + sugar + phosphate). Bennu samples contain nucleobases^47^, including the five canonical nucleobases that comprise nucleic acids in terrestrial biology. Minerals that catalyse the sugar ribose (C_5_H_10_O_5_) include hydroxyapatite^49^, and salts can catalyse the formation of nucleosides (nucleobase + sugar)^50^. Although both phosphate and salts are observed in Bennu samples, experimental studies are needed to determine whether nucleotides could have formed in the chemical conditions on Bennu’s parent body. If nucleotides form, polymers of 30–60 monomers can be catalysed by montmorillonite^51^, although such a process has not been demonstrated for the phyllosilicate phases in Bennu samples. Finally, as polymers increase in length, adsorption strength to the mineral surface increases^52^. These polymers could be released by concentrated salt solutions. Such processes have yet to be studied or their products observed in Bennu samples; nevertheless, the presence of clays, phosphates and evaporites indicative of alkaline fluids makes Bennu a promising target for investigation of potential prebiotic organic synthesis.

## Methods

### Samples

The samples studied were derived from two sources: spillover on the avionics deck, outside the spacecraft’s touch-and-go sample acquisition mechanism (TAGSAM)^53^, and the bulk sample within TAGSAM itself. Samples from the avionics deck were part of the ‘quick-look’ phase of preliminary examination^5,54^ and are denoted OREX-5#####-#; TAGSAM samples are denoted OREX-8#####-#. All samples studied are aggregates of particles less than a few millimetres in the longest dimension^5^. Samples were curated in high-purity nitrogen at NASA’s Johnson Space Center, shipped in containers either sealed in a nitrogen glove box or flushed with nitrogen and, except as noted, stored in nitrogen in the laboratories of individual analysts. Even with handling in air during sample preparation and equipment loading, most samples had been exposed to air for only hours to days. For complete details of sample handling following return, see ref. ^5^.

### Optical microscopy

Reflected-light images of unprepared particles were acquired using a Keyence VHX-7000 4 K digital microscope in the Kuiper-Arizona Laboratory for Astromaterials Analysis (K-ALFAA) at the University of Arizona.

### SEM

At the Natural History Museum in London, rough, coated particles were analysed at 6 kV in high vacuum using a FEI Quanta 650 field emission (FE)-SEM equipped with a Bruker QUANTAX EDS system and an annular, high-sensitivity XFlash FlatQUAD silicon-drift detector (SDD). They were then embedded in epoxy, polished, carbon coated and analysed using a ZEISS Ultra plus FE-SEM and Oxford Instruments AZtec EDS system with a conventional, large-area Ultim Max 170 SDD. An intermediate accelerating voltage of 10 kV was applied to limit the interaction volume of emitted X-rays to the submicrometre scale. Hyperspectral imaging datasets, providing complete spectra for each pixel of the SEM image, were acquired of whole particles using automated stage control at pixel resolutions of 200–300 nm.

At the Smithsonian Institution’s National Museum of Natural History in Washington, DC, fine to coarse particles were mounted on iridium-coated Parafilm. then coated with iridium and analysed at 15 kV and around 0.5 nA in high vacuum using a Thermo Fisher Quattro FE-SEM equipped with two opposing Thermo Fisher UltraDry 60 mm^2^ EDS detectors. Elemental maps were acquired by both detectors at roughly 1 µm per pixel using Thermo Fisher Pathfinder software. Both large-area detectors were used simultaneously to acquire fast maps with high counts, and to mitigate certain shadowing issues when analysing rough samples. Maps of loose grains were investigated, then used to inform sectioning. Samples were then mounted in epoxy, cut in half, remounted and polished. Polished sections were coated with iridium and analysed at 15 kV and about 0.5 nA. Some images and spectra of particles and polished samples were acquired at 7 kV, to reduce interaction volume.

At the Johnson Space Center, sample nos. OREX-803065-0 and OREX-803174-0 were characterized using a JEOL 7600 F FE-SEM equipped with a 170-mm^2^ SSD type Oxford Instruments Ultim Max EDS detector. Particles were attached to an Al cylinder SEM mount using double-sided C tape. Following initial documentation by optical microscopy, samples were sputter coated with about 5 nm of C to assist with charge dissipation during SEM analysis. Oxford AZtec ‘Point & ID’ and ‘Mapping’ software was used for imaging, point spectra, element mapping and data reduction. Characterization of regions of interest was performed at an accelerating voltage of 15 kV using both secondary electron and low-angle BSE imaging modes. EDS spectra were acquired at 15 kV, with acquisition times ranging from 20 to 200 s and an incident beam current of approximately 900 pA. As with point spectra, EDS element maps (1,024 × 768 pixels) were acquired at 15 kV and magnification ranging from ×1,500 to ×10,000, with a dwell time of 400 ms and three to five frame accumulations. To confirm light element identification, some point analyses were also reacquired at an accelerating voltage of 5 kV.

At Curtin University, a 5-nm evaporative carbon coat was applied before characterization by secondary electron and BSE imaging, electron backscatter diffraction (EBSD) and EDS mapping, using a Tescan MIRA3 VP FE-SEM fitted with an Oxford Instruments AZtec v.5.1 Symmetry EBSD–EDS acquisition system at the John de Laeter Centre (JdLC). Secondary electron and BSE imaging was performed using 5 kV acceleration voltage. EBSD data were collected using 20-kV acceleration voltage, roughly 1.5-nA beam current, 70° stage tilt, map step size 60 nm, halite match units and 15-ms frame acquisition time. EBSD data were processed using AZtecCrystal 2.1 software, including removal of isolated misindexed points and zero-solution extrapolation to six nearest neighbours.

### HR-CL

HR-CL was performed using a MonoCL4 GATAN system and a high-sensitivity photomultiplier mounted on a field emission gun SEM (JEOL JSM7000F) at Centre de Recherches sur l’HétéroEpitaxie et ses Applications, Valbonne, France. Panchromatic images were acquired using a high-sensitivity photomultiplier with wavelength sensitivity ranging from 190 to 900 nm. The image of OREX-800045-106 was acquired at room temperature and 5 keV, with a current of 4 nA and pixel time of 800 µs.

### EMPA

Electron microprobe analysis was conducted on Ir-coated specimens using the JEOL 8530 F+ Hyperprobe Field Emission Electron Probe Microanalyzer at the Smithsonian Institution. Carbonate analyses were run at 15 kV and 10 nA, with an analytical spot size of 5 µm. Fe and Mn were analysed using a LIFL crystal, Mg using a TAPL crystal and Ca using a PETL crystal. Standard analyses were performed on magnetite (USNM 114887), calcite (USNM 13621), dolomite (USNM 10057), siderite (R-2460) and rhodonite. Secondary standardization was conducted using calcite, dolomite and rhodochrosite; analyses of magnetite and olivine were run under the same conditions. Analyses were conducted at 15 kV and 10 nA, with an analytical spot size of 1 µm. Standard analyses were performed on chromite (USNM 117075), ilmenite (USNM 96189), magnetite (USNM 114887), manganite (USNM 157872), bytownite (R-2912), forsterite (P140), San Carlos olivine (USNM 111312/444; Fo90) and Springwater olivine (USNM 2566; Fo83). Secondary standardization was conducted using magnetite, San Carlos olivine and Springwater olivine.

EMPA analyses were carried out using a Cameca SX-100 electron microprobe located at K-ALFAA, University of Arizona. To prevent charging of the sample during EMP analysis, the section was coated with a thin film of carbon (20 nm). Wavelength-dispersive X-ray spectroscopy analyses of Mg,Na phosphate were conducted using a 1-µm beam size, acceleration voltage of 15 kV and beam current of 8 nA. The standards used for Mg,Na phosphate were fluorapatite (F, P, Ca), Fo_92_ olivine (Si, Mg), rhodonite (Mg), fayalite (Fe), anorthite (Al), baryte (S), potassium feldspar (K) and scapolite (Cl). For carbonates, the standards used were albite (Na), Fo_92_ olivine (Si), dolomite (Mg), calcite (Ca), Mn carbonate (Mn), apatite (P), baryte (S) and fayalite (Fe).

### FIB-SEM

At the University of Arizona, an electron-transparent (under 100 nm) section of Na carbonate grains was prepared using the Thermo Fisher Scientific Helios G^3^ FIB-SEM, located at K-ALFAA. We followed previously described procedures for the production of electron-transparent cross-sections of fine grains (see, for example, ref. ^55^). Briefly, we deposited a 12-μm-wide × 4-μm-tall protective capping layer of C on top of a vein containing Na carbonate grains. We used a standard stair-step cut to create the lamellae, extracted it in situ and thinned it to electron transparency at 30 keV and currents ranging from 2.5 to 0.79 nA.

At Curtin University, three 20–50-μm opaque grey particles (OREX-501070-0) were manually separated from 1 mg of aggregate fines collected from the avionics deck (OREX-501021-0) for quick-look analysis, mounted on a carbon tab followed by the application of a 5-nm evaporative carbon coat. Particles were ion milled parallel to the substrate with a Ga^+^ FIB to show particle interiors using a Tescan LYRA dual-beam FIB-SEM in the JdLC at Curtin University, Australia (20231115_FIB-SEM_CUWA_OREX-501070-0_1). Initially, 30-kV ion beam energy was used for FIB milling, with a final low-energy step at 5 kV to remove surface damage from milling. The particle was reimaged 8 months following initial FIB milling and storage in an autodesiccator at relative humidity 30–40%, demonstrating that halite-sylvite grains had disappeared entirely (20240715_FIB-SEM_CUWA_OREX-501021-0_1).

### TEM

At the University of Arizona, characterization of the FIB section was performed using the 200-keV Hitachi HF5000 STEM located at K-ALFAA. This unit is equipped with a cold-field emission gun; a third-order spherical-aberration corrector for STEM mode, bright-field, dark-field and secondary electron STEM detectors; an Oxford Instruments X-Max N 100 TLE EDS system with dual 100-μm^2^ windowless SDDs providing a solid angle (*Ω*) of 2.0 sr; and a post-column Gatan Quantum EELS instrument. TEM images and electron-diffraction patterns were acquired with a Gatan OneView 4,000 × 4,000-pixel complementary metal oxide semiconductor camera.

The sample was imaged in, and chemically mapped in, STEM mode (converged beam) using EDS. EDS spectrum images were acquired at 136-pm probe size and 512 × 512 pixels over a 20-keV energy range with 2,048 channels, a process time setting of 3 (roughly equivalent to a time constant) and a frame time of 8 μs. Areas of the FIB section were analysed in TEM mode (parallel illumination) for grain size and crystallinity. Bright-field TEM images and SAED patterns were acquired from regions of interest. SAED patterns were measured using the Crystallographic Image Processing Software Package^56^ based on calibrated camera constants. EELS spectrum images were acquired in high-angle EELS mode (approximately 60-mrad collection angle) using a 136-pm probe, a 5-mm spectrometer-entrance aperture, drift tube offset of 270 eV, dispersion of 0.05 eV per channel (around 102.4-eV energy range), varied rectilinear map size (for example, 33 × 43 pixels), step size of 10–15 nm and acquisition times of 0.002 s for the low-loss region and 0.5 s for the high-loss region. We used a drift tube offset of 270 eV and dispersion of 0.05 eV ch^−1^ to evaluate the C–K energy-loss near-edge structure (ELNES). The various allotropes of C have distinct ELNES^57^, and so the ELNES of the suspected Na carbonate was used to confirm the presence of the carbonate anion.

At the Schwiete Cosmochemistry Laboratory of Goethe University Frankfurt, we prepared fine-particle samples for TEM analysis by depositing crushed powders onto TEM copper mesh grids with a lacey carbon support. We stored the grids in a desiccator and used gentle plasma cleaning just before TEM analyses. TEM data were collected on a Thermo Scientific Talos F200-X G2 STEM, operated at 200 kV. A Ceta-S 4,000 × 4,000 16 M camera was used for imaging, and four windowless SDDs for EDS analyses.

At Lawrence Berkeley National Laboratory, microscopy was carried out on the FEI TitanX TEM at the Molecular Foundry, supported by the Office of Science, Office of Basic Energy Sciences, of the US Department of Energy under contract no. DE-AC02-05CH11231. Imaging was performed in STEM configuration with a high-angle, annular dark-field detector at 300 keV.

### XRD

Three white particles picked out from the aggregate sample allocated to the Natural History Museum were mounted on a carbon adhesive disc, and confirmed as Mg,Na phosphate using an FEI Quanta 650 SEM-EDS (OREX-800032-110). The mineralogy and crystalline structure of one of the particles (approximately 100 × 200 μm^2^) was then characterized using micro-XRD. For this, the particle was removed from the carbon disc using a needle and fixed, using a small amount of glue, to a Kapton loop mount. The mount was then attached to an adjustable goniometer and centred in the primary X-ray beam of a Rigaku Rapid II microdiffractometer equipped with a curved, two-dimensional imaging plate detector. The size of the primary X-ray beam was restricted to around 100 μm using a collimator, and micro-XRD patterns were acquired from the particle with Cu radiation.

### Scanning transmission X-ray microscopy

Scanning transmission X-ray measurements were carried out at Beamline 5.3.2.2 of the Advanced Light Source at Lawrence Berkeley National Laboratory, supported by the Office of Science, Office of Basic Energy Sciences, of the US Department of Energy under contract no. DE-AC02-05CH11231. X-ray absorption near-edge stacks were acquired across the C–K edge with 100-nm pixels at 278–360 eV, with an energy step of 0.1 eV or higher. Data were processed using Labview and Python. Na_2_CO_3_ and NaHCO_3_ evaporite standards were produced by drop casting a DI solution of each salt onto a TEM grid. Following evaporation, carbonate peak positions were measured and then fit using a linear background and a Gaussian; the Gaussian centroid was taken as the position of the carbonate peak. Bennu sample measurements were performed in the same fashion for direct comparison. Beamline C–K edge energy was calibrated using the 292.74 eV, 1–3-s peak of CO_2_ gas^58^. In addition, energy calibration was internally verified for each experimental measurement using the *I*_0_ (incident photon flux) minimum. Any calibration drift regardless of cause, whether within a single measurement or between measurements, would show as a drift in the *I*_0_ minimum. The *I*_0_ minimum was measured at 284.99–285.06 eV for all datasets, demonstrating that energy calibration was stable and that all spectra can be compared directly with a beamline tolerance below 0.1 eV.

### ToF–SIMS

ToF–SIMS mapping was performed using an IONTOF M6 instrument at JdLC, and data were processed using SurfaceLab 7 software (20231125_SIMS_CUWA_OREX-501070-0_1). Areas of interest were presputtered using a 1-kV O_2_^+^ beam for 30 s at 400 nA current. ToF–SIMS mapping utilized a 60-keV Bi_3_^++^ primary beam in positive and negative ion mode, with 0.2–0.5-pA analysis current, in rapid imaging mode (unbunched) and delayed extraction mass analyser setting. Data were collected with a mass/charge range of 0–155 Da and effective mass resolution of above 5,000. Mass accuracy and detection limits were typically under 1 ppm for halogens and volatile metal ions of interest. Map pixel size was 58 nm. The Na number of halite-sylvite was calculated from ToF–SIMS data as a ratio of combined detected peak intensities using positive ions (positive ion Na number = 100 × (Na^+^ + Na_2_Cl^+^/(Na^+^ + Na_2_Cl^+^ + K^+^ + K_2_Cl^+^))) and negative ions (negative ion Na number = 100 × (Na^−^ + NaCl^−^ + NaCl_2_^−^/(Na^−^ + NaCl^−^ + NaCl_2_^−^ + K^−^ + KCl^−^ + KCl_2_^−^)).

### Evaporation calculations

The degree of evaporation can be calculated from the salinity of an evaporating fluid. Modelling of Searles Lake brines^4^ yields an approximate salinity for precipitation of Na,Ca carbonate of 220, Na carbonate of 330 and Na chloride of 360 (g kg^−1^). In the absence of detailed knowledge of the fluid on Bennu’s parent body, we estimate salinity from Cl concentration and water:rock ratio. The average CI chondrite chlorine concentration of 630 ppm (ref. ^59^) and a water:rock ratio by mass of 0.5 (ref. ^60^) yields an approximate initial salinity of 125 (4× terrestrial seawater). We recognize the uncertainty in this value, because allowable water:rock mass ratios (0.5–1.0 (ref. ^60^)), whether Cl was completely dissolved in the fluid, and concentrations of other ions (for example, Na, Mg) are needed to fully define initial salinity. Using salinity of 125, formation of Na,Ca carbonate occurs at around 45% evaporation, precipitation of Na carbonate at about 60% evaporation and halite at 65% evaporation. The initial salinity used in the calculation is probably close to an upper limit, resulting in these calculated degrees of evaporation being the probable lower limits of actual evaporation.

## Online content

Any methods, additional references, Nature Portfolio reporting summaries, source data, extended data, supplementary information, acknowledgements, peer review information; details of author contributions and competing interests; and statements of data and code availability are available at 10.1038/s41586-024-08495-6.

## Figures and Tables

**Extended Data Table 1:**
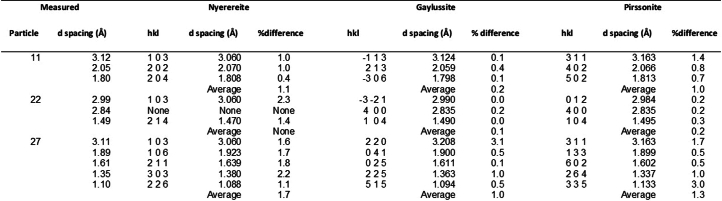
Measured *d*-spacing (Å) for three Na,Ca carbonates from OREX-800045-102

The anhydrous Na,Ca carbonate nyerereite and the hydrous Na,Ca carbonates gaylussite and pirssonite are shown for comparison.

**Extended Data Table 2:**
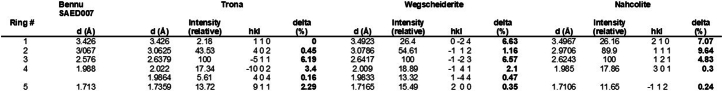
Measured *d*-spacing (angstroms) for Na carbonate from OREX-803088-100

The H-bearing or hydrous carbonates trona, wegscheiderite, and nahcolite are shown for comparison.

**Extended Data Table 3:**
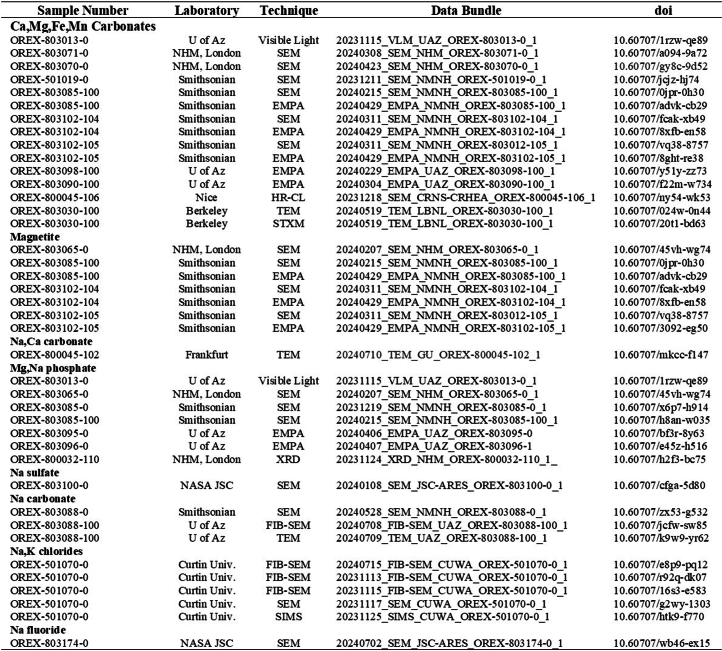
Sample numbers, analytical details, data bundle identifier, and doi for evaporite phases

Data is available from Astromat (astromat.org).

## Data Availability

Instrument data supporting the experimental results from the samples analysed in this study will be available from Astromat (astromat.org) at the DOIs listed in Extended Data Table 3.

## References

[1] Tutolo, B. M. & Tosca, N. J. Dry, salty, and habitable: the science of alkaline lakes. *Elements* **19**, 10–14 (2023).

[2] Postberg, F. et al. Sodium in salts in E-ring ice grains from an ocean below the surface of Enceladus. *Nature* **459**, 1098–1101 (2009). 19553992 10.1038/nature08046

[3] De Sanctis, M. C. et al. Bright carbonate deposits as evidence of aqueous alteration on (1) Ceres. *Nature* **536**, 54–57 (2016). 27362221 10.1038/nature18290

[4] Olson, K. J. & Lowenstein, T. K. Searles Lake evaporite sequences: indicators of late Pleistocene/Holocene lake temperatures, brine evolution, and pCO_2_. *GSA Bull.* **133**, 2319–2334 (2021).

[5] Lauretta, D. S. et al. Asteroid (101955) Bennu in the laboratory: properties of the sample collected by OSIRIS-REx. *Meteorit. Planet. Sci.* **59**, 2453–2486 (2024).

[6] Bada, J. L. How life began on Earth: a status report. *Earth Planet. Sci. Lett.* **226**, 1–15 (2024).

[7] Bowell, R. J., Lagos, L., de los Hoyos, C. R. & Declerco, J. Classification and characteristics of natural lithium resources. *Elements* **16**, 259–264 (2020).

[8] Mancinelli, R. L., Fahlen, T. F., Landheim, R. & Klovstad, M. R. Brines and evaporites: analogs for Martian life. *Adv. Space Res.* **33**, 1244–1246 (2004).

[9] Hansen, C. J. et al. Enceladus’ water vapor plume. *Science* **311**, 1423–1425 (2006). 10.1126/science.1121254 16527971

[10] Treiman, A. H. The nakhlite meteorites: augite-rich igneous rocks from Mars. *Geochemistry* **65**, 203–270 (2005).

[11] Zolensky, M. E. et al. Asteroidal water within fluid inclusion-bearing halite in an H5 chondrite, Monahans (1998). *Science* **285**, 1377–1379 (1999). 10464091 10.1126/science.285.5432.1377

[12] Walsh, K. J. et al. Numerical simulations suggest asteroids (101955) Bennu and (162173) Ryugu are likely second or later generation rubble piles. *Nat. Commun.* **15**, 5653 (2024). 38969628 10.1038/s41467-024-49310-0 PMC11226714

[13] Lauretta, D. S. et al. The OSIRIS-REx target asteroid (101955) Bennu: constraints on its physical, geological, and dynamical nature from astronomical observations. *Meteorit. Planet. Sci.* **50**, 834–849 (2015).

[14] DellaGiustina, D. N. et al. Variations in color and reflectance on the surface of asteroid (101955) Bennu. *Science* **370**, eabc3660 (2020). 33033157 10.1126/science.abc3660

[15] Rozitis, B. et al. Asteroid (101955) Bennu’s weak boulders and thermally anomalous equator. *Sci. Adv.* **6**, eabc3699 (2020). 33033037 10.1126/sciadv.abc3699 PMC7544501

[16] Lauretta, D. S. et al. Spacecraft sample collection and subsurface excavation of asteroid (101955) Bennu. *Science* **377**, 285–291 (2022). 35857591 10.1126/science.abm1018

[17] Jawin, E. R. et al. Boulder diversity in the nightingale region of asteroid (101955) Bennu and predictions for physical properties of the OSIRIS‐REx sample. *J. Geophys. Res. Planet.* **128**, e2023JE008019 (2023).

[18] Stroud, R. M. et al. Electron microscopy observations of the diversity of Ryugu organic matter and its relationship to minerals at the micro‐ to nano‐scale. *Meteorit. Planet. Sci.* **59**, 2023–2043 (2024).

[19] Gainsforth, Z. et al. Coevolution of phyllosilicate, carbon, sulfide, and apatite in Ryugu’s parent body. *Meteorit. Planet. Sci.* **59**, 2073–2096 (2024).

[20] Barber, D. J. Matrix phyllosilicates and associated minerals in C2M carbonaceous chondrites. *Geochim. Cosmochim. Acta* **45**, 945–970 (1981).

[21] DuFresne, E. R. & Anders, E. On the chemical evolution of carbonaceous chondrites. *Geochim. Cosmochim. Acta* **26**, 1085–1114 (1962).

[22] Jenkins, L. E. et al. Winchcombe: an example of rapid terrestrial alteration of a CM chondrite. *Meteorit. Planet. Sci.* **59**, 988–1005 (2024).

[23] Gounelle, M. & Zolensky, M. E. A terrestrial origin for sulfate veins in CI1 chondrites. *Meteorit. Planet. Sci.* **36**, 1321–1329 (2001).

[24] Braukmüller, N., Wombacher, F., Hezel, D. C., Escoube, R. & Müller, C. The chemical composition of carbonaceous chondrites: implications for volatile element depletion, complementarity and alteration. *Geochim. Cosmochim. Acta* **239**, 17–48 (2018).

[25] Hardie, L. A. & Eugster, H. P. The evolution of closed-basin brines. In Morgan, B. A. (ed.) *Fiftieth Anniversary Symposia: Mineralogy and Petrology of the Upper Mantle, Sulfides, Mineralogy and Geochemistry of Non-Marine Evaporites: Mineralogical Society of America Special Paper 3*, 273–290 (Mineralogical Society of America, 1970).

[26] Tosca, N. J. & Tutolo, B. M. How to make an alkaline lake: fifty years of chemical divides. *Elements* **19**, 15–21 (2023).

[27] Smith, G. I. & Stuiver, M. Subsurface stratigraphy and geochemistry of late Quaternary evaporites, Searles Lake, California. *Geol. Surv. Prof. Pap.* **1043**, 1–130 (1979).

[28] Naseem, M. et al. Salt distribution from freezing intrusions in ice shells on ocean worlds: application to Europa. *Planet. Sci*. *J*. **4**, 181 (2023).

[29] Sridhar, S., Bryson, J. F. J., King, A. J. & Harrison, R. J. Constraints on the ice composition of carbonaceous chondrites from their magnetic mineralogy. *Earth Planet. Sci. Lett.* **576**, 117243 (2021).

[30] White, L. et al. Evidence for sodium-rich alkaline water in the Tagish Lake parent body and implications for amino acid synthesis and racemization. *Proc. Natl Acad. Sci. USA* **117**, 11217–11219 (2020). 32393617 10.1073/pnas.2003276117 PMC7260959

[31] Kampf, A. R., Mills, S. J., Nash, B. P., Jensen, M. & Nikischer, T. Apexite, NaMg(PO_4_)·9H_2_O, a new struvite-type phase with a heteropolyhedral cluster. *Am. Min.* **100**, 2695–2701 (2015).

[32] Postberg, F. et al. Detection of phosphates originating from Enceladus’s ocean. *Nature* **618**, 489–493 (2023). 37316718 10.1038/s41586-023-05987-9 PMC10266972

[33] Köningsberger, E., Köningsberger, L.-C. & Gamsjäger, H. Low-temperature thermodynamic model for the system Na_2_CO_3_-MgCO_3_-CaCO_3_-H_2_O. *Geochim. Cosmochim. Acta* **63**, 3105–3119 (1999).

[34] Jagniecki, E. A., Jenkins, D. M., Lowenstein, T. K. & Carroll, A. R. Experimental study of shortite (Na_2_Ca_2_(CO_3_)_3_) formation and application to the burial history of the Wilkins Peak Member, Green River Basin, Wyoming, USA. *Geochim. Cosmochim. Acta* **115**, 31–45 (2013).

[35] Jagniecki, E. A., Lowenstein, T. K., Jenkins, D. M. & Demicco, R. V. Eocene atmospheric CO_2_ from the nahcolite proxy. *Geology* **43**, 1075–1078 (2015).

[36] Fox-Powell, M. G. & Cousins, C. R. Partitioning of crystalline and amorphous phases during freezing of simulated Enceladus ocean fluids. *J. Geophys. Res. Planets* **126**, e2020JE006628 (2021).

[37] Ishimaru, K. & Lauretta, D. S. Analysis of layered boulders on asteroid (101955) Bennu and their implications for fluid flow on the parent body. *Meteorit. Planet. Sci.* **59**, 193–210 (2023).

[38] Kaplan, H. H. et al. Bright carbonate veins on asteroid (101955) Bennu: implications for aqueous alteration history. *Science* **370**, eabc3557 (2020). 33033155 10.1126/science.abc3557

[39] National Academies of Sciences, Engineering, and Medicine. *Origins, Worlds, and Life: A Decadal Strategy for Planetary Science and Astrobiology 2023–2032* (The National Academies Press, 2023).

[40] Nakamura, T. et al. Formation and evolution of carbonaceous asteroid Ryugu: direct evidence from returned samples. *Science* **379**, eabn8671 (2022). 10.1126/science.abn8671 36137011

[41] Matsumoto, T. et al. Sodium carbonates on Ryugu as evidence of highly saline water in the outer Solar System. *Nat. Astron.*10.1038/s41550-024-02418-1 (2024).

[42] Yoshimura, T. et al. Chemical evolution of primordial salts and organic sulfur molecules in the asteroid 162173 Ryugu. *Nat. Commun.* **14**, 5284 (2023). 37723151 10.1038/s41467-023-40871-0 PMC10507048

[43] Raponi, A. et al. Mineralogy of Occator Crater on Ceres and insight into its evolution from the properties of carbonates, phyllosilicates, and chlorides. *Icarus* **320**, 83–96 (2019).

[44] De Sanctis, M. C. et al. Fresh emplacement of hydrated sodium chloride on Ceres from ascending slaty fluids. *Nat. Astron.* **4**, 786–793 (2020).

[45] Raymond, C. A. et al. Impact-drive mobilization of deep crustal brines on dwarf planet Ceres. *Nat. Astron* **4**, 741–747 (2020).

[46] McCord, T. B. et al. Thermal and radiation stability of the hydrated salt minerals epsomite, mirabilite, and natron under Europa environmental conditions. *J. Geophys. Res.* **106**, 3311–3319 (2001).

[47] Glavin, D. P. et al. Abundant ammonia and nitrogen-rich soluble organic matter in samples from asteroid (101955) Bennu. *Nat. Astron.*10.1038/s41550-024-02472-9 (2025). 10.1038/s41550-024-02472-9 PMC11842271 39990238

[48] Spencer, J. R. et al. Cassini encounters Enceladus: background and the discovery of a south polar hot spot. *Science* **311**, 1401–1405 (2006). 16527965 10.1126/science.1121661

[49] Usami, K. & Okamoto, A. Hydroxyapatite: catalyst for a one-pot pentose formation. *Org. Biomol. Chem.* **15**, 8888–8893 (2017). 28952648 10.1039/c7ob02051a

[50] Fuller, W. D., Sanchez, R. A. & Orgel, L. E. Studies in prebiotic synthesis. VII. Solid-state synthesis of purine nucleosides. *J. Mol. Evol.* **1**, 249–257 (1972). 4681226 10.1007/BF01660244

[51] Ferris, J. P., Hill, A. R. Jr., Liu, R. & Orgel, L. E. Synthesis of long prebiotic oligomers on mineral surfaces. *Nature* **318**, 59–61 (1996). 10.1038/381059a0 8609988

[52] Hill, A. R., Böhler, C. & Orgel, L. E. Polymerization on the rocks: negatively charged α-amino acids. *Orig. Life Evol. Biosph.* **28**, 235–243 (2001). 10.1023/a:1006572112311 9611764

[53] Bierhaus, E. B. et al. The OSIRIS-REx spacecraft and the touch-and-go sample acquisition mechanism (TAGSAM). *Space Sci. Rev.* **214**, 107 (2018).

[54] Lauretta, D. S. et al. OSIRIS-REx sample analysis plan – revision 3.0. Preprint at 10.48550/arXiv.2308.11794 (2023).

[55] Zega, T. J., Haenecour, P. & Floss, C. An in situ investigation on the origins and processing of circumstellar oxide and silicate grains in carbonaceous chondrites. *Meteorit. Planet. Sci.* **55**, 1207–1227 (2020).

[56] Hovmöller, S. CRISP: crystallographic image processing on a personal computer. *Ultramicroscopy* **41**, 121–135 (1992).

[57] Garvie, L. A. J., Craven, A. J. & Brydson, R. Use of electron-energy loss near-edge fine structure in the study of minerals. *Am. Min.* **79**, 411–425 (1994).

[58] Prince, K. C., Avaldi, L., Coreno, M., Camilloni, R. & de Simone, M. Vibrational structure of core to Rydberg state excitations of carbon dioxide and dinitrogen oxide. *J. Phys. B At. Mol. Opt. Phys.* **32**, 2551 (1999).

[59] Palme, H. & Zipfel, J. The composition of CI chondrites and their contents of chlorine and bromine: results from instrumental neutron activation analysis. *Meteorit. Planet. Sci.* **57**, 317–333 (2022).

[60] Russell, S. S., Suttle, M. D. & King, A. J. Abundance and importance of petrological type 1 chondritic material. *Meteorit. Planet. Sci.* **57**, 277–301 (2022).

